# Electromechanical and scar characteristics at left ventricular lead implant site in the context of overall dyssynchrony with cine DENSE predict cardiac resynchronization therapy outcomes

**DOI:** 10.1186/1532-429X-16-S1-O53

**Published:** 2014-01-16

**Authors:** Kenneth C Bilchick, Sujith Kuruvilla, Yasmin S Hamirani, Raghav Ramachandran, Samantha Clarke, Sophia Cui, Michael Salerno, Jeffrey Holmes, Christopher M Kramer, Frederick H Epstein

**Affiliations:** 1 University of Virginia Health System, Charlottesville, Virginia, USA

## Background

In clinical patients undergoing cardiac resynchronization therapy (CRT), myocardial properties (mechanical, electrical, and scar-mediated) at the left ventricular (LV) lead position (LVLP) and overall LV dyssynchrony (circumferential uniformity ratio estimate [CURE, 0-1]) are of interest with respect to CRT outcomes. We sought to define the relative importance of these factors for CRT response in a clinical CMR study with longitudinal follow-up.

## Methods

**Figure 1:**
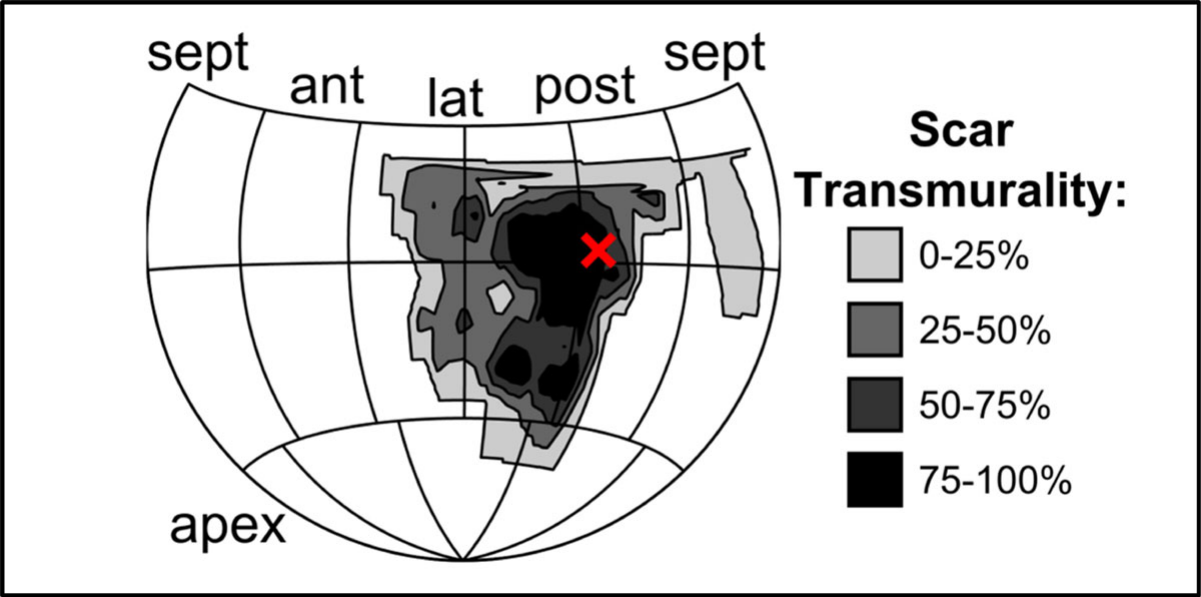
**LV Lead Position in Scar in a Patient with Prior Myocardial Infarction**.

All patients had CMR including cine displacement encoding with stimulated echoes (DENSE) for strain imaging and late gadolinium enhancement (LGE) and echocardiography prior to CRT. Electrical timing at the LVLP (LV lead electrical delay [LVLED, 0-1]) was assessed during the procedure. CRT response was defined as a 15% reduction in the LV end-systolic volume on follow-up echocardiography at 6 months. Cine DENSE was performed in 4 short-axis and 3 long-axis slices with displacement encoding applied in two orthogonal in-plane directions for each slice (TR/TE 17 ms/1.9 ms, slice thickness 8 mm, field of view 350 × 350 mm, flip angle 15 degrees, pixel size 2.8 × 2.8 mm, fat suppression, and displacement-encoding frequency 0.1 cycles/mm). LV scar at the LVLP was determined quantitatively based on fluoroscopic images (Figure 1).

## Results

**Figure 2:**
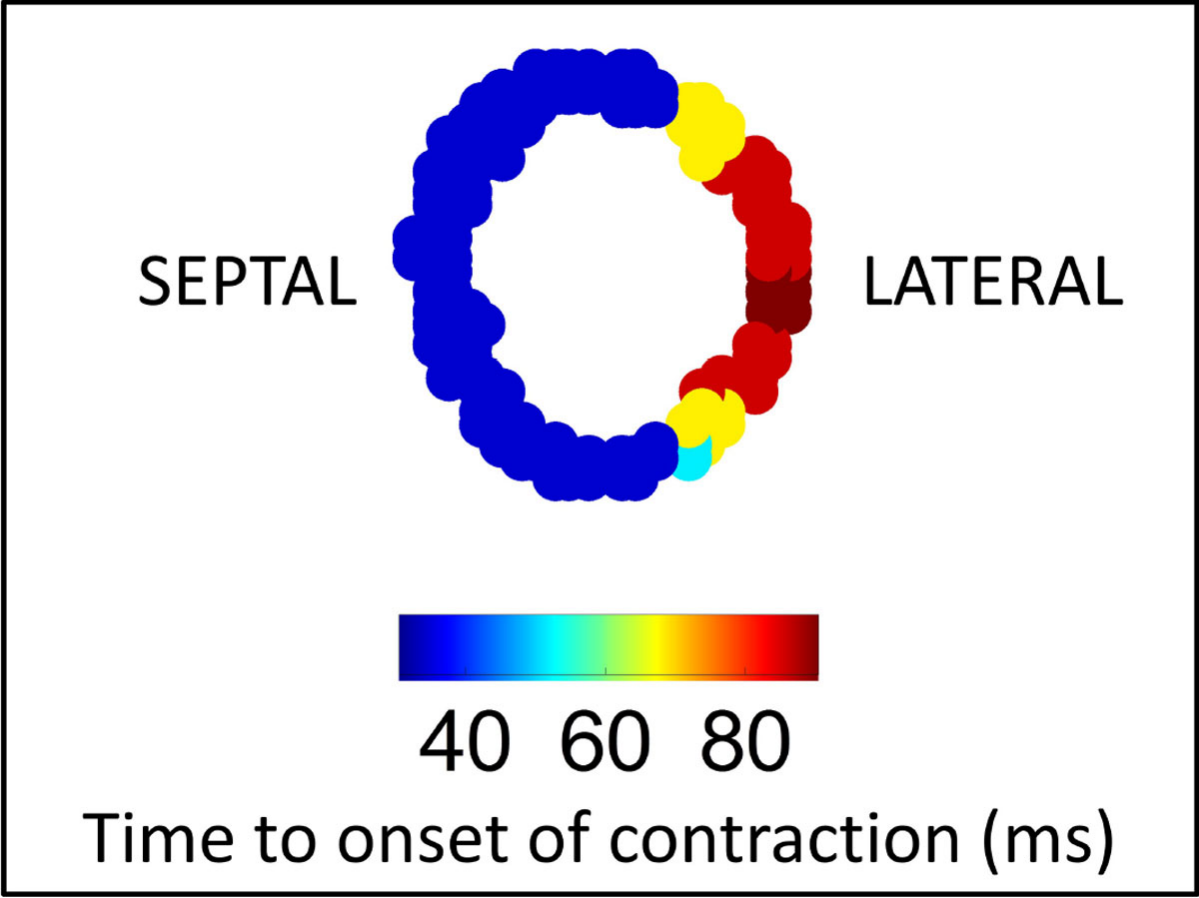
**Activation Map for Onset of Contraction (Ecc) in a Patient with Left Bundle Branch Block**.

Of the 75 patients studied (median age 65.9 years [IQR 57.8-74.3], 25% female), 40 (53%) were CRT responders. 16 (21.3%) died, 20 (26.7%) had a heart failure hospitalization, and 12 (16.0%) had sustained ventricular tachycardia or fibrillation during a median follow-up of 2.6 years. High quality Ecc was obtained with DENSE, as shown in the activation map (Figure 2) for onset of contraction in a patient with left bundle branch block (LBBB). Based on the DENSE strain imaging, we developed a highly predictive multivariable logistic regression model for CRT response (AUC = 0.946; p < 0.0001) with CURE for overall dyssynchrony (OR 2.9 per 0.1 decrease in CURE, 95% C.I. 1.7-4.9), delayed onset of Ecc at the LVLP (OR 7.1 [1.2-41.2]), absence of scar at the LVLP (OR 3.4 [2.3-77.0]), and electrical timing at the LVLP based on the LVLED (OR 1.7 [1.1-2.5] per 0.1 increase in LVLED). Early LV stretch at the LVLP, defined as a delay in the time to a negative slope in the Ecc curve, was more predictive of CRT response (p = 0.03) than time to peak strain at the LVLP (p > 0.5). Of note, the corresponding multivariable linear model was highly predictive of the percent change in LVESV after CRT (R2 = 0.61). The multivariable model with CURE ≥ 0.70 (HR 4.19, 95% C.I. 1.51-11.6) and LVLED ≤0.50 (HR 4.92, 95% C.I. 1.71-14.0) was highly predictive of death, while CURE ≥ 0.60 predicted sustained ventricular tachycardia (HR 8.24, 95% C.I. 1.06-63.9).

## Conclusions

Mechanical, electrical, and scar properties at the LVLP and overall mechanical dyssynchrony based on CURE provide highly accurate prediction of LV reverse remodeling after CRT. CMR DENSE and LGE are very useful for pre-CRT assessment of heart failure patients.

## Funding

This research was supported by Dr. Bilchick's NIH K23 grant HL094761 and Dr. Holmes' R01 grant HL085160.

